# Treatment of Early Childhood Medulloblastoma by Postoperative Chemotherapy Alone—A Critical Review

**DOI:** 10.4137/cmo.s392

**Published:** 2009-03-04

**Authors:** Ricardo J. Komotar, Marc L. Otten, Matthew C. Garrett, Richard C.E. Anderson

**Affiliations:** Department of Neurosurgery Columbia University New York, NY.

Despite comprehensive treatment with surgery, radiation, and chemotherapy, medulloblastoma remains the most common malignant brain tumor in children with a five-year disease free survival rate of only 40% in those less than 10 years of age. Although adjuvant regimens are an integral component in patient management, radiotherapy in very young children is limited by the susceptibility of the immature brain to radiation-induced cognitive deficits. Thus, the development of an effective adjuvant protocol employing chemotherapy alone is of substantial interest.

Rutkowski and colleagues published the results of their trial investigating the treatment of medulloblastoma solely with postoperative chemotherapy (*Rutkowski* et al. *Treatment of Early Childhood Medulloblastoma by Postoperative Chemotherapy Alone. N Engl J Med. 2005;352(10): 978–986)*. In this multi-institutional clinical trial, 43 patients with histologically confirmed medulloblastoma were treated after surgery with an intensive chemotherapy protocol, consisting of three cycles of intravenous cyclophosphamide, vincristine, methotrexate, carboplatin, and etoposide, as well as intraventricular methotrexate. Results were as follows: in children with complete resection, partial resection, and macroscopic metastases, the five-year progression-free and overall survival rates were 82% and 93%, 50% and 56%, and 33% and 38%, respectively. Furthermore, desmoplastic histology, metastatic disease, and age less than two years were found to be independent prognostic factors for tumor relapse and poor survival. Of note, there were no instances of unexpected toxicity, with only asymptomatic leukoencephalopathy occurring in the majority of patients. Although the mean IQ of patients in this cohort was significantly lower than that of healthy, matched controls, it was higher than that of patients receiving cerebral radiation. Based on these findings, the authors concluded that in young children without metastases, chemotherapy may be used alone as adjuvant treatment for medulloblastoma, especially after gross total resection or those with desmoplastic variants, which portends a more favorable prognosis.

This article highlights the growing importance of chemotherapy in the treatment of pediatric brain tumors, and represents a substantial step forward in the management of medulloblastoma. In the past, postoperative radiotherapy was frequently used in this patient population. Although highly effective, the risk of permanent cognitive side effects severely limits its use in children younger than five years of age. In such cases, chemotherapy may be employed to delay neuraxis irradiation. This study, which is the largest conducted to date in children <3 years old, demonstrates that patients receiving intensive chemotherapy may not only achieve excellent outcomes, but may also avoid the need for radiotherapy all together. As expected, cognitive testing in this cohort demonstrated milder side effects from chemotherapy than radiotherapy. It is important to note, however, factors other than radiation therapy may impact on quality of life and neuropsychiatric development. Regardless, the authors’ findings have implications for the current management guidelines of pediatric CNS malignancies by supporting the notion that radiotherapy may be avoided in young children without compromising prognosis.

## Figures and Tables

**Figure 1.**
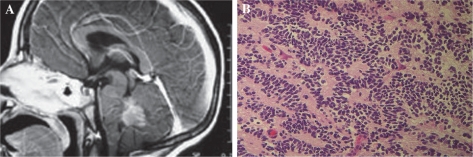
**A)** Sagittal T1-weighted post-contrast MRI demonstrating a medulloblastoma filling the 4th ventricle. **B)** Histopathology of a medulloblastoma demonstrating densely packed cells forming Homer Wright rosettes.

